# P-201. Mental Health Interventions in TB Care: A Systematic Scoping Review in the SAARC Region

**DOI:** 10.1093/ofid/ofaf695.423

**Published:** 2026-01-11

**Authors:** Namrata Rana, Pushpita Samina, Gurher Sidhu, Amrita Daftary

**Affiliations:** Rowan-Virtua School of Osteopathic Medicine, Stratford, NewJersey; McMaster University, Toronto, Ontario, Canada; York University, Toronto, Ontario, Canada; York University, Toronto, Ontario, Canada

## Abstract

**Background:**

One in four people with tuberculosis (TB) face mental health (MH) challenges. Poor MH hinders TB health-seeking, treatment success, and post-treatment outcomes. We reviewed approaches to provide MH care for people with TB in the South Asian Association for Regional Cooperation (SAARC) region, where 37% of people with TB live.

**Figure:**
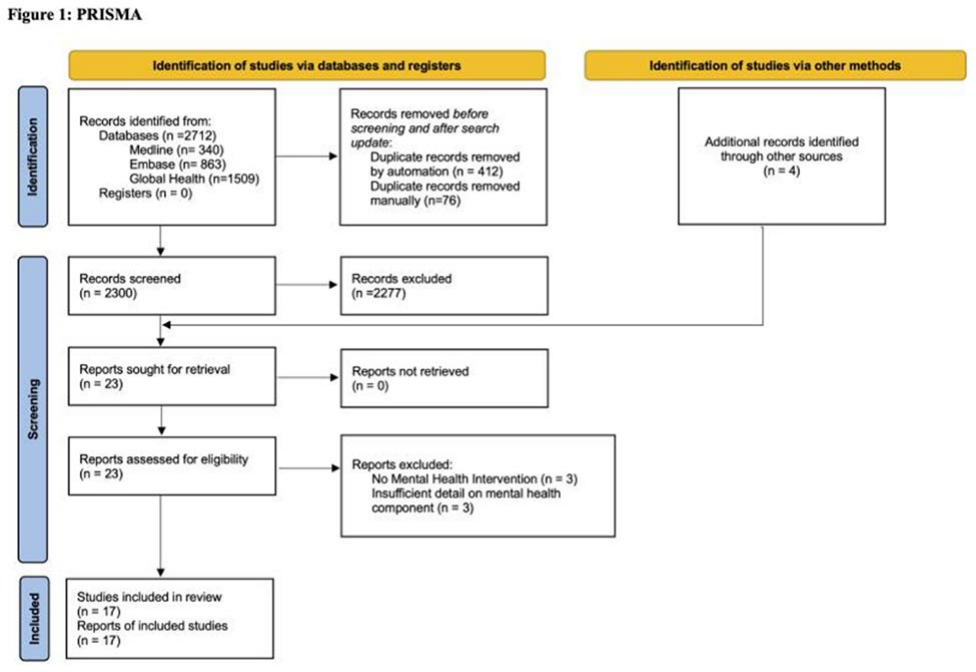

**Methods:**

We searched three databases (Medline, Embase, Global Health) for studies implementing an explicit mental health service or intervention to people undergoing TB screening, diagnosis and/or treatment in a SAARC country from 2000-2024. Extracted data were narratively synthesized.

**Results:**

2304 unique records were retrieved and yielded 17 eligible studies from India (n=10), Pakistan (n=6), and Nepal (n=1). Eight studies were among people with MDRTB. Interventions included MH counselling (n=15, varied schedule, content, framework, and delivery approach), pharmacological intervention (n=9, anti-depressants, anxiolytics, withholding MDRTB drugs) and breathing exercises (n=1). Interventions were implemented at clinics providing TB services (n=15) with off-site activities (n=5) and/or referrals to mental health centres (n=4); one was entirely via telehealth. MH professionals (psychiatrists, psychologists, n=9), nurses (n=1), social workers (n=3), pharmacists (n=1), and other cadres (peer supporters, patient navigators, lay counsellors, DOTS facilitators, n=9) were engaged in intervention delivery. Mental health was assessed using standardized tools (n=14); five studies assessed substance use. All studies reporting post-intervention mental health outcomes (n=7) (anxiety, depression) and TB outcomes (n=10) (case notifications, treatment completion, adherence, failure, loss-to-follow-up, drug resistance, death) reported improved outcomes. Improved patient satisfaction (n=2), quality of life (n=2), cardio-pulmonary measures (n=1), and reduced stigma (n=1) were also reported. Seven studies implemented cointerventions: nutrition (n=5), economic support (n=7), and vocational rehabilitation (n=2).

**Conclusion:**

Effective people-centred TB care requires addressing TB-comorbidities. We found MH interventions can be integrated in TB care settings to support achievement of improved TB and MH outcomes

**Disclosures:**

All Authors: No reported disclosures

